# Removal and Biodegradation of 17β-Estradiol and Diethylstilbestrol by the Freshwater Microalgae *Raphidocelis subcapitata*

**DOI:** 10.3390/ijerph15030452

**Published:** 2018-03-05

**Authors:** Weijie Liu, Qi Chen, Ning He, Kaifeng Sun, Dong Sun, Xiaoqing Wu, Shunshan Duan

**Affiliations:** 1 Institute of Hydrobiology, Jinan University, Guangzhou 510632, China; liuweijie_84@163.com (W.L.); cq92088@outlook.com (Q.C.); jnu_sundong@163.com (D.S.); 2 Key Laboratory of Aquatic Eutrophication and Control of Harmful Algal Blooms, Guangdong Higher Education Institutes, Guangzhou 510632, China; 3 Collage of Life Science and Resources and Environment, Yichun University, Yichun 336000, China; hening2010@163.com; 4 Research Center of Offshore Marine Environment, South China Institute of Environmental Sciences, MEP, Guangzhou 510655, China; jnuskf@163.com (K.S.); wxqingAnn@163.com (X.W.)

**Keywords:** 17β-estradiol, diethylstilbestrol, removal, biodegradation, *Raphidocelis subcapitata*

## Abstract

Natural steroidal and synthetic non-steroidal estrogens such as 17β-estradiol (E2) and diethylstilbestrol (DES) have been found in natural water, which can potentially endanger public health and aquatic ecosystems. The removal and biodegradation of E2 and DES by *Raphidocelis subcapitata* were studied in bacteria-free cultures exposed to single and mixture treatments at different concentrations for 96 h. The results showed that *R. subcapitata* exhibited a rapid and strong ability to remove E2 and DES in both single and mixture treatments by biodegradation. At the end of 96 h, the removal percentage of single E2 and DES achieved 82.0%, 80.4%, 74.6% and 89.9%, 73.4%, 54.1% in 0.1, 0.5, and 1.5 mg·L^−1^, respectively. With the exception of the 0.1 mg·L^−1^ treatment at 96 h, the removal capacity of E2 was more efficient than that of DES by *R. subcapitata*. Furthermore, the removal percentage of mixture E2 and DES achieved 88.5%, 82.9%, 84.3% and 87.2%, 71.8%, 51.1% in 0.1, 0.5, and 1.5 mg·L^−1^, respectively. The removal percentage of mixed E2 was significantly higher than that of the single E2. The presence of DES could accelerate the removal of E2 from the mixture treatments in equal concentrations. In addition, the removal was mainly attributed to the biodegradation or biotransformation process by the microalgae cells rather than simple sorption and accumulation in the cells. The microalgae *R. subcapitata* demonstrated a high capability for the removal of the E2 and DES indicating future prospects for its application.

## 1. Introduction

Natural and synthetic estrogens are commonly found in wastewater and natural water, which can disrupt the endocrine function in wildlife and human beings [1]. The former are contributed predominantly by humans and livestock excretions [2] including estrone (E1), 17β-estradiol (E2), and estriol (E3). Synthetic estrogens are divided into synthetic steroidal estrogens and synthetic non-steroidal estrogens. Synthetic steroidal estrogens are extensively used as a constituent of contraceptives such as ethinyloestradiol (EE2). Diethylstilbestrol (DES), as a typical synthetic non-steroidal estrogen, was prescribed to millions of pregnant women to prevent miscarriages and other pregnancy complications from 1940 to 1970 [3,4]. Additionally, DES was banned in 1972 as a human pharmaceutical due to its adverse effects on the female reproductive tracts [5]. However, DES is still used as a growth promoter in terrestrial livestock or fish in some parts of the world including China [6]. Estrogens are also released into the environment through direct discharge, sewage treatment plants, production wastes, and discarded products [7,8]. The occurrence and distribution of estrogens such as E1, E2, EE2, and DES have been widely reported in the aquatic environment. According to reports from recent years, the detected concentration of these estrogens ranged from 1.75 to 560 ng·L^−1^ in E1, from 1.31 to 130 ng·L^−1^ in E2, from 0.27 to 170 ng·L^−1^ in EE2, and from 2.54 to 6.75 ng·L^−1^ in DES [9,10,11,12]. The effects of estrogenic activity include imposex, feminization, and the disruption of the normal function of the endocrine [13]. Although the detected estrogen concentrations were ng·L^−1^ levels [12], they can have detrimental effects at extremely low concentrations (<1 ng·L^−1^) [14]. Moreover, estrogens can accumulate through the food chain and the effects can possibly appear in subsequent generations, thereby endangering public health and the sustainable development of humans [15].

The removal and biodegradation of organic contaminants by microalgae have been reported. Green microalgae such as *Chlorella* species have been reported to remove inorganic nutrients, heavy metals, and toxic organic contaminants from wastewater with low cost and high efficiency [16]. Some green microalgae have been shown to have the ability to remove and degrade steroid hormones [17]. For instance, *Chlorella vulgaris* has been reported to remove EE2 and E1, and *Scenedesmus dimorphus* has also been used to remove E1, E2, E3, and 17α-estradiol in batch culture [18,19]. *Chlamydomonas reinhardtii* presented high adsorption percentages of 86% and 71% for E2 and EE2. Nearly half of the removal was attributed to biodegradation processes, while the rest of the removal was due to adsorption [20]. Furthermore, contaminants have been removed by microalgae due to biodegradation or biotransformation rather than simple adsorption on the algal cell surface [21]. Moreover, a mixed microalgae culture model was tested for use in the removal and biodegradation of complex and persistent contaminants [22]. In nature water, estrogens are frequently present as a mixture containing different natural and synthetic estrogens. The mixture can have different fates in wastewater treatment plants and in rivers or lakes [17]. However, previous studies have been more focused on a single estrogen, and the removal of a mixture of estrogens from aquatic environments by microalgae has been less reported. Additionally, studies about DES biodegradation by microalgae are also rare.

Reports have shown that bacteria live freely attached to the algal surface or as intracellular algal co-existing in microalgae cultures [23]. Interactions of bacteria and microalgae include symbiosis, commensalism, mutualism, parasitism and competition, not only beneficial trophic relationships, but also negative effects such as the inhibition of algal growth or lysing the cells. Although some bacteria communities associated with microalgae can be tuned to specialize in the degradation of specific pollutants [24], bacteria can stimulate, inhibit, kill or alter phytoplankton physiology. The elimination of bacteria can better realize the ability of microalgae in contaminant removal. Therefore, the removal of bacteria from stock microalgae cultures would provide a simpler system for the research of estrogen removal and biodegradation by microalgae.

*Selenastrum capricornutum*, *Pseudokirchneriella subcapitata* and *Raphidocelis subcapitata* are the same species named in different periods [25]. The species is a sickle-shaped and unicellular green microalga that is normally found in fresh water. It has frequently been used for ecotoxicological tests for decades and used for removing organic contaminants in recent years [26]. *R. subcapitata* have been reported to remove and degrade phenanthrene (PHE), fluoranthene (FLA), and pyrene (PYR) using monooxygenase and dioxygenase enzymatic pathways [27]. It has also been reported to degrade benzo(a)pyrene (BaP) to *cis*-dihydrodiols by a dioxygenase enzyme system [26] and to remove parent PAHs via a cytochrome P-450 system [28]. The ability of nonylphenol (NP) biodegradation by *R. subcapitata* has also been reported [29]. Thus, it is possible that this species has the potential to remove estrogens. The present study aimed to investigate the response and tolerance ability of *R. subcapitata* exposure on 17β-estradiol (E2) and diethylstilbestrol (DES) and to examine the natural steroidal estrogen and synthetic non-steroidal estrogen removal and biodegradation capacity of *R. subcapitata* including single and mixture treatments.

## 2. Materials and Methods

### 2.1. Microalgae Species and Culture Conditions

*Raphidocelis subcapitata* was obtained from the Algae Culture Collection of the Department of Ecology in Jinan University (Guangzhou, China). The algae were cultivated with a BG11 medium in a homoeothermic incubator (FPG-3, Ningbo, China) at 60 μmol·m^−1^·s^−1^, a diurnal cycle of 12 h light and 12 h dark and a temperature 25 ± 1 °C. The components of the BG11 medium were as follows: NaNO_3_ 1.5 g·L^−1^, K_2_HPO_4_ 40 mg·L^−1^, CaCl_2_·2H_2_O 36 mg·L^−1^, MgSO_4_·7H_2_O 75 mg·L^−1^, NaHCO_3_ 20 mg·L^−1^, citric acid 6 mg·L^−1^, and ferric ammonium citrate 6 mg·L^−1^. The trace metal solution contained H_3_BO_3_·2.86 mg·L^−1^, MnCl_2_·4H_2_O 1.81 mg·L^−1^, ZnSO_4_·7H_2_O 222 mg·L^−1^, Na_2_MoO_4_·2H_2_O 390 mg·L^−1^, CuSO_4_·5H_2_O 79 mg·L^−1^, and Co(NO_3_)_2_·6H_2_O 49.4 mg·L^−1^ [30].

### 2.2. Removal of Bacteria from Algal Cultures

Based on a previous report [31], mid-exponential phase *R. subcapitata* cultures (100 mL) were filtered with a 10 µm pore size membrane. The algal cells were suspended in a 50 mL BG11 medium which was autoclaved at 121 °C for 15 min before centrifuging (3000 rpm, 10 min) and washed three times. The washed cells were suspended in a 50 mL sterile BG11 medium and treated with 0.005% Tween-80 and 0.1 M EDTA for 1 h at 20 °C. Next, lysozyme (0.5 mg·mL^−1^) was added for further treatment (10 min at 20 °C) before sodium dodecyl sulfate (SDS, 0.25%) was added into the culture environment. After 10 min of SDS treatment time, the algal cells were centrifuged (3000 rpm, 10 min) and washed twice to remove the lysozyme and SDS, and then suspended in a 50 mL BG11 medium. A cocktail of antibiotics including 100 µg·mL^−1^ penicillin and 50 µg·mL^−1^ kanamycin was added to the cultures. The treated *R. subcapitata* was cultured in the incubator at 20 ± 1 °C with a diurnal cycle of 12 h light and 12 h dark for 7 days. An assessment for bacterial presence was carried out after sub culturing three times to remove the antibiotics. The axenic status of *R. subcapitata* was confirmed after sub culturing three times in a sterile BG11 medium without antibiotics. A 4′,6-diamidino-2-phenylindole (DAPI) stain was added into samples at a final concentration of 4 µg·L^−1^ and then cultured in the dark for 15 min at 4 °C. Cells were filtrated with black polycarbonate membranes (0.22 μm pore size, Whatman, Shanghai, China). Bacterial presence was immediately examined by epifluorescence microscopy (BX41, Olympus, Tokyo, Japan) using oil immersion.

### 2.3. Growth Inhibition Test

17β-Estradiol (E2) and diethylstilbestrol (DES), purchased from Sigma-Aldrich (Shanghai, China) were dissolved in acetone and the dissolved form was added to a stock solution at a concentration of 1 g·L^−1^. An initial growth culture was prepared 5 days before the tests to ensure the population was in the exponential growth phase at the beginning of the test [32]. All solutions and experimental containers were autoclaved at 121 °C for 15 min. The tests (in triplicates) used 250 mL Erlenmeyer flasks filled with 100 mL of the sterile BG11 medium. The stock culture of each treatment yielded the following concentrations: 0, 0.1, 0.5, 0.8, 1.5, and 3.2 mg·L^−1^ of E2 and 0, 0.1, 0.3, 0.5, 0.9, and 1.5 mg·L^−1^ of DES. The mixed E2 and DES treatment was 0, 0.1, 0.3, 0.5, 1.5, and 3 mg·L^−1^, and the mass concentration rate of E2:DES was 1:1 based on a previous report [33]. The initial cell density was 0.1 × 10^5^ cells·mL^−1^. All flasks were randomly arranged in racks. To avoid microalgae sedimentation, all flasks were hand-mixed twice per day during the test. Treatment with an equivalent amount of acetone (0.01%, *v*/*v*) was included as a control. After 24, 48, 72, and 96 h of exposure time, 2 mL of each sample were fixed in 0.5% of methanal to further measure the algal density by counting with a Neubauer’s chamber (Purity, Beijing, China) under microscope (Eclipse50i, Nikon, Tokyo, Japan).

For the cell dry weight measurement, the pore-size GF/F glass-fiber filter (0.45 μm, Whatman, Shanghai, China) was dried overnight in an oven (DHG-9140A, Jinghong, China) at 60 °C until a constant weight was reached. A 20 mL culture was filtered through the filters. The filters with algal cells were dried overnight in an oven at the same conditions above until a constant weight was reached. The difference between the final weight and the weight before filtration was the dry weight of the algal cells.

According to the Organization for Economic Co-operation and Development (OECD) (2006) [34], the 96 h EC_50_ (a concentration that causes a 50% reduction of algal growth compared to the negative control) was calculated using linear regressions of the inhibition rate and expressed in terms of the estrogen concentration (mg·L^−1^).

### 2.4. Residual Estrogens

Immediately after the sampling timepoint, microalgal cells were separated from 5 mL medium by centrifugation at 5000 × rpm for 15 min at 4 °C. E2 and DES in the aqueous supernatant were extracted twice with ethyl acetate (25 mL) by liquid-liquid microextraction (DLLME), and 4-nonylphenol (4-NP) at a final concentration of 1 mg L^−1^ was the internal surrogate standard, as described by Wang et al. [17], with some modifications. The extracts were evaporated to 1 mL by using a rotary evaporator and transferred into a reaction vial with 100 μL (1:1) *N*,*O*-bis(trimethylsilyl)trifluoroacetamide (BSTFA, Sigma-Aldrich, St. Louis, MO, USA) as the derivatization in a water bath at 70 °C for 1 h. Then, the reaction system was dried with a gentle nitrogen stream at 25 °C and dissolved in 100 μL of ethyl acetate for further analysis by gas chromatography-mass spectrometry (GC-MS).

### 2.5. Estrogens Adsorbed onto Cell Surface

For every estrogens concentration, blank groups without algae were set up to evaluate the variation in estrogens concentration under abiotic conditions. The microalgal cells from the above section were washed with 5 mL ethyl acetate and shaken for 60 s, then the E2 and DES contained in the solution was considered as the surface adsorbed estrogens and then extracted with DLLME as described above and analyzed with GC-MS.

### 2.6. Estrogens Absorbed into Cells

After adding the appropriate amount of anhydrous Na_2_SO_4_, the microalgae cells obtained from the above section were mixed with ethyl acetate (4 mL) and sonicated for 30 min, then, the sample was centrifuged for 5 min at 5000 rpm. The cell pellets were extracted three times and the solvent fractions were combined for further analysis with GC-MS. Based on the measured concentrations, the removal efficiency (*R*) and biodegradation percentage (*BDP*) of estrogens by the algal biomass were calculated as previously described with minor modifications according to the following equations:

(1) $$R=100\;\times \left(C_{i}-\;C_{f}\right)/C_{i}$$

*R* is the dissolved estrogens removal rate (percent); *C_i_* and *C_f_* are the initial and final concentrations (mg·L^−1^) of estrogens in the solution, respectively, and:

(2) $$\mathrm{BDP}\;\left(100\%\right)=\;100\times \left(C_{i}-C_{r}-AL-C_{c}\times W_{a}-C_{d}\times W_{a}\right)/C_{i}$$

where *C_i_* is the initial concentration in the solution; *C_r_* is the residual concentration in the solution; *AL* is the concentration of abiotic losses (mg·L^−1^); *C_d_* is the concentration (mg·g^−1^) dry weight of estrogens adsorbed on the cell wall; *C_c_* is the concentration (mg·g^−1^ dry weight) of estrogens accumulated in the algal cells; and *W_a_* is the dry weight of algal biomass expressed in g·L^−1^ [16].

### 2.7. Determination of Estrogens

The analysis and quantification of estrogens in the extract were determined using a GC-MS (Agilent 7890/5975CMSD) with HP-5MS fused silica capillary column (Agilent, Santa Clara, CA, USA; 60 m × 0.25 mm × 0.25 μm). The carrier gas was helium and set at a constant flow rate of 1 mL·min^−1^. The GC injector port was held isothermally at 280 °C. The mass selective detector (MSD) was operated both in scan and selected ion monitoring (SIM) mode. The GC column temperature program was as follows: the initial oven temperature was set at 100 °C and increased to 300 °C at a rate of 10 °C·min^−1^ with a holding of 3 min at 300 °C. The split less injection was 1 µL and solvent delay was 6 min. The limit of quantification for both E2 and DES was 0.01 µg·L^−1^. The results obtained were compared to those obtained from a control group without estrogens.

### 2.8. Statistical Analysis

Statistical analysis was carried out using the SPSS16.0 package (SPSS Inc., Chicago, IL, USA). One Way-ANOVA followed by the Duncan test was used to check the significance of the treatments. The significance of difference among the E2 and DES in single and mixture treatments at the same concentration was comparatively analyzed with a one-way ANOVA. Levels of significance used were *p* < 0.05 and were described as “significant”. Data are presented as the mean ± standard deviation (mean ± SD) unless otherwise stated.

## 3. Results

### 3.1. Growth of R. subcapitata Exposed to Estrogens

**Figure 1:**
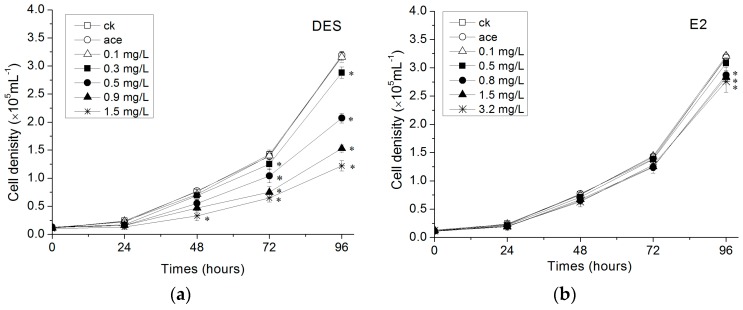

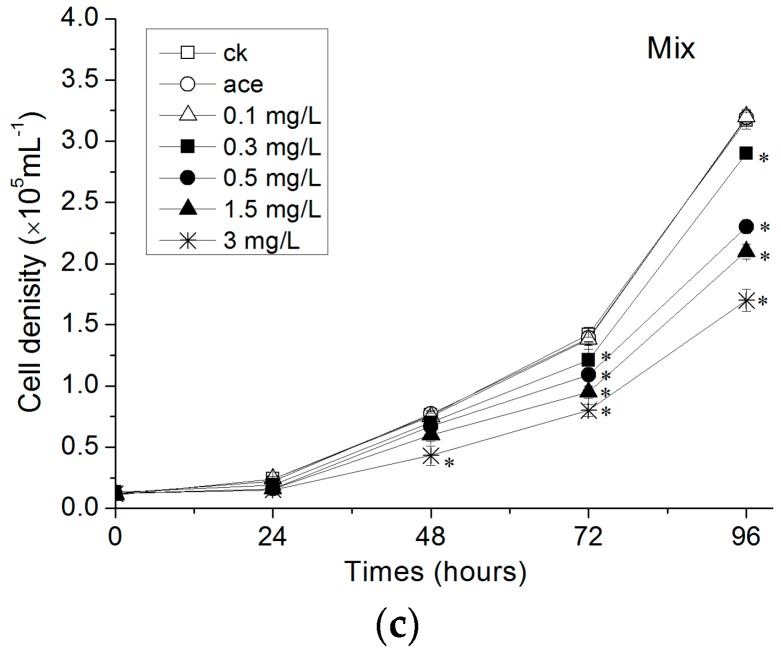
Effect of DES (**a**), E2 (**b**) and Mixture (**c**) treatments at concentrations on the cell number of *R. subcapitata,* values are the mean ± standard deviation (SD) (*n* = 3). The “ck” represents control check and “ace” represents acetone treatment. An asterisk (*) indicates a value significantly different from control (*p* < 0.05).

The 96 h EC50 of *R. subcapitata* exposure to estrogens is shown in Table 1 and the growth of *R. subcapitata* exposure to single and mixtures of E2 and DES are shown in Figure 1. The solvent (acetone) in the designated concentration in the study (0.05%) had no obvious effect on *R. subcapitata* growth, excluding the possibility of the solvent to have caused toxic effects on algae. Results showed a negative effect with the test concentrations of DES. Growth of *R. subcapitata* was significantly reduced at 1.5 mg·L^−1^ after 48 h exposure with a reduction of 56.6%. After 72 h exposure, the growth had a strong decrease at 0.3–1.5 mg·L^−1^ concentration treatments. At the end of 96 h, growth of *R. subcapitata* at 0.3–1.5 mg·L^−1^ concentration treatments were further decreased and the reduction of 1.5 mg·L^−1^ concentration treatments was 61.5%. Compared with the DES treatments, the growth of *R. subcapitata* exposed to E2 were reduced gradually. Cell abundance had a significant decrease for 96 h exposure at 0.8–3.2 mg·L^−1^ concentrations. When the microalgae cells were exposed to mixtures of estrogen (1:1) for 48 h, the growth was obviously decreased at a 3 mg·L^−1^ concentration with the reduction was 43.4%. With the exposure time increasing to 72 and 96 h, significant decreases in growth were also observed in mixture treatments at 0.3–3 mg·L^−1^ concentrations. 

### 3.2. Removal of Estrogens by R. subcapitata

#### 3.2.1. Abiotic Losses of Estrogens (AL)

**Figure 2:**
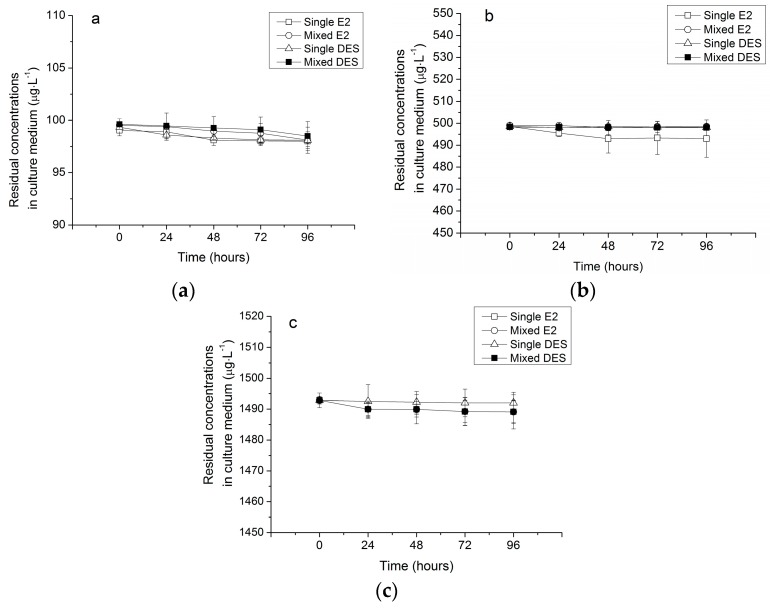
Residual concentrations of E2 and DES in culture medium without microalgae: (**a**) 0.1 mg·L^−1^; (**b**) 0.5 mg·L^−1^L; and (**c**) 1.5 mg·L^−1^initial spiked values. Values are the mean ± standard deviation (SD) (*n* = 3).

In the control without microalgae, the residual concentrations of single E2 and DES, as well as the residual concentrations of E2 and DES in a mixture, that remained in the medium were the same as the initial spiked values at 0.1, 0.5, and 1.5 mg·L^−1^ for 96 h exposure, indicating that abiotic losses was negligible and would not influence the evaluation of the capacity of *R. subcapitata* in the removal of contaminants (Figure 2).

#### 3.2.2. Removal of Estrogens in Single Treatments by *R. subcapitata*

After exposure in single estrogens for 96 h, the residual, intracellular, and extracellular E2 and DES contents of *R. subcapitata* under different treatments are shown in Table 2. The amounts of residual estrogens in the medium all decreased gradually within 96 h. In the 0.1 mg·L^−1^ concentration treatments, the residual amount of E2 was less than DES at 24 h and 48 h (12% and 10%, respectively). After the 96 h treatment, the residual amount of E2 was more than DES at 7.7%. In the 0.5 and 1.5 mg·L^−1^ treatments, the residual amount of E2 was much less than DES at 24 h, 48 h and 96 h (11.1%, 7.4%, 6.7% and 7.4%, 18.4%, 20.1%, respectively). The amount of estrogen uptake included that adsorbed onto the cell surfaces (extracellular) and absorbed into the cells (intracellular). The amounts of extracellular E2 and DES were increased with concentrations of exposure during 96 h. The amount of extracellular E2 was less than DES except the 0.1 mg·L^−1^ concentration treatment at 96 h. Intracellular estrogens were also increased with exposure concentrations. The amount of intracellular E2 was less than that of DES at the 0.1 mg·L^−1^ treatment and became more significant at 0.5 mg·L^−1^ treatments. With the exception of the 0.1 mg·L^−1^ treatment, *R. subcapitata* could remove more of E2 than DES and more DES were uptaken by the microalgae than that of E2.

#### 3.2.3. Removal of Estrogens in Mixed Treatments by *R. subcapitata*

The residual and uptake of mixed estrogens under different treatments after 96 h exposure are shown in Table 3. There were no significant differences between the residual amounts of E2 and DES in the mixed treatment at 0.1 mg·L^−1^ concentration. While the amounts of E2 remaining in the medium were reduced more than DES in the 0.5 and 1.5 mg·L^−1^ treatments at the end of 24 h, 48 h, and 96 h (3.6%, 6.9%, 10.7% and 14.7%, 21.4%, 32.7%, respectively). The amount of extracellular E2 was less obvious than DES in all concentrations except the 0.1 mg·L^−1^ treatment at the first 24 h. Furthermore, the intracellular E2 in the mixture treatments were also less than DES and the statistical significance were observed other than that in 0.1 mg·L^−1^ treatment at the first 24 h.

According to Table 2 and Table 3, the residual amounts of E2 were less than that of DES in both the single and mixture treatments except at the 0.1 mg·L^−1^ concentration. The concentrations of both extra- and intra-cellular E2 were also less than that of DES in the single and mixed. Moreover, the residual amounts of E2 in the mixed treatments were less than that of E2 in the single treatment after 96 h. 

### 3.3. Removal Efficiency and Biodegradation of Estrogens by R. subcapitata

**Figure 3:**
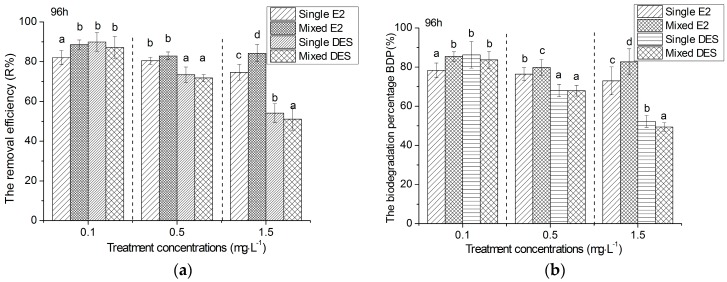
Removal efficiency (**a**) and biodegradation (**b**) of estrogens by *R. subcapitata* in different concentrations after 96 h. Mean and standard deviation of the three replicates are shown. The mean with lowercase a–c in each concentration indicates that they were significantly different at *p* ≤ 0.05 according to one-way ANOVA test (Duncan).

The removal efficiency and biodegradation of E2 and DES were calculated to directly show the capacity of *R. subcapitata* for removing estrogens. Figure 3 shows the removal efficiency and biodegradation of estrogens by *R. subcapitata* in different treatments with 0.1, 0.5, 1.5 mg·L^−1^ concentrations at the end of 96 h. Significance of difference among the E2 and DES in single and mixture treatments at the same concentration were comparatively analyzed with a one-way ANOVA followed by the Duncan test. After exposure for 96 h, the removal efficiency of single E2 treatments was not only obviously less than E2 in mixture, but also significant less than DES in both the single and mixture treatments at 0.1 mg·L^−1^. In the 0.1 mg·L^−1^ treatment, the removal efficiency of E2 were all higher than that of DES in both the single and mixture treatments. When the exposure concentration was increased to 1.5 mg·L^−1^, the removal efficiency of E2 in the mixture was significant higher than the single and mixed DES, and even that of E2 in a single exposure. Moreover, the removal efficiency of the single E2 was also higher than that of DES in the single and mixture treatments. The trend of estrogen biodegradation was general as the removal efficiency except 0.5 mg·L^−1^. At the 0.5 mg·L^−1^ concentration treatment, the biodegradation of E2 in mixture was obviously higher than that of E2 in single exposure.

## 4. Discussion

### 4.1. Influence of Estrogens on R. subcapitata Growth

The present study revealed that not only DES could inhibit the growth of *R. subcapitata*, but that E2 could also cause a significant decline in the growth at 0.8 mg·L^−1^ after 96 h exposure, indicating that estrogens could cause an adverse effect on *R. subcapitata* cells. Microalgae can be influenced by contaminants; however, the sensibility and tolerance are related to contaminant compounds and are species-dependent. E2 was slightly toxic to *R. subcapitata*, it was reported that could even be tolerated at concentrations up to 10 mg·L^−1^ [33]. However, E2 has been also reported where the 96 h EC_50_ was 0.87 and 1.01 mg·L^−1^ for *Pseudokirchneriella subcapitata* (the same as *R. subcapitata*) [35]. There was the difference of 96 h EC_50_ of E2 among the reports, even in the present study. It may cause by some reasons. On one hand, the microalgae cultured by different conditions such as the medium, light intensity, temperature, pH and light-dark cycle could make the different growth situation and adaptive features. These differences may cause the different in accumulation of the intracellular organic matters and extracellular secondary metabolites [36], and it could be one of the reasons that made different sensibility and tolerance of the same specie microalgae under exposure to containment. On the other hand, in our study, removal of bacteria from algal cultures was performed to evaluate the ability of the algae to remove estrogens. In the process, some antibiotics were added. This may be enhanced the tolerance of *R. subcapitata* to estrogens [37]. 

Compared to E2, DES has high toxicity to *R. subcapitata* in the present study. As an inhibitor of the plasma membrane H^+^-ATPase, DES has been reported to block proton (H^+^) transport [38] and inhibit cell division in plant cells [39], indicating that DES could cause more damage to microalgae. This also agreed with the previous finding that DES could decrease growth by inhibiting the glucose activation of *Dunaliella* [40]. When *R. subcapitata* was exposed to mixed estrogens after 96 h, the toxicology was less than the exposure to a single DES, but more toxic than being exposed to a single E2. That may indicate that E2 could decrease the toxic effects of DES to the microalgae. Additionally, it has been reported that E2 can improve antioxidative enzyme activities and avoid H_2_O_2_ generation to reduce oxidative stress injury from heavy metal induced [41]. Furthermore, E2 can also disturb the expression of phenylpropanoid-flavonoid pathway genes and decrease the accumulation of phenols, flavonoids, and anthocyanins of plant cells [42]. For microalgae, there are also several mechanisms to protect themselves from the toxicity of contaminants [43]. These responses include detoxification, antioxidant defense mechanisms, and some unknown pathways [44,45]. In addition, some species of microalgae can metabolize certain endocrine disrupting chemicals to an intermediate with no estrogenic activity or less toxic metabolite [46,47]. The different tolerance of microalgae to estrogens might mean a difference in efficiency and response to exposure stress. Although E2 and DES could influence the growth of *R. subcapitata*, the toxicity concentration was more than 0.5 mg·L^−1^, indicating the strain was able to remove estrogens with high tolerance.

### 4.2. Capacity and Mechanism of R. subcapitata for the Removal of Estrogens

The removal of synthetic organic compounds by a variety of microalgae species has been widely demonstrated. In this study, the removal capacity of estrogens E2 and DES in single and mixture by microalgae *R. subcapitata* was assessed. From the results, the removal efficiency of E2 was generally higher than DES in single treatments. The tests showed that about 74.6% and 54.1% of E2 and DES, respectively were removed from culture after 96 h in single treatments. The biodegradation percentage (BDP) was about 72.9% and 52.3% in the single E2 and DES treatments, respectively. As a natural steroidal estrogen, E2 was easier to remove by the microalgae than other synthetic steroidal estrogens such as 17α-ethynylestradiol (EE2) [17]. Several studies about the removal of E2 by microalgae were reported and demonstrated that *Chlorella vulgaris*, *Anabaena cylindrical*, *Spirulina platensis*, and *Scenedesmus quadricauda* could effectively remove E2 from culture and could accelerate its removal from the wastewater [18,48]. Due to its persistent pollution characteristics, DES is more difficult to degrade than steroidal estrogens. The removal mechanisms by microalgae are three processes involving bio-adsorption (including passive adsorption and active absorption), bioaccumulation, and biodegradation [49]. Compared with other steroidal estrogens, DES was more readily adsorbed and more difficult to degrade [50,51]. In this study, extracellular and intracellular E2 and DES were compared. The extracellular DES was more than the E2 adsorbed by the cell walls in the single and mixture treatments. According to the reports, the ability of the microalgae cells to accumulate contaminants was highly correlated with the cell volume and surface area, and freshwater algae with its high surface area to volume ratio showed a high potential for sorption and interaction with organic contaminants [52,53]. Moreover, physiological characteristics and enzymes involved in endocrine disrupting contaminant degradation might also be important in determining the difference in the removal of E2 and DES [54]. A previous report found that the toxic contaminant removal capacity of the cell walls was less than that of the cell contents [55]. In the present study, it agreed that E2 of extracellular concentrations were more than that of the intracellular. However, the concentrations of extracellular and intracellular DES all exhibited different tendencies. Concentrations of DES adsorbed by the cell walls were not less than that of the cell contents. Although the bioaccumulation of DES was more than E2, in the current set of experiments, bioadsorption and bioaccumulation still had negligible contributions on the total removal of estrogens, which suggests that the major mechanism of estrogen removal by *R. subcapitata* was biodegradation. This was consistent with the results from previous reports on the removal of carbamazepine, nonlyphone, and steroid estrogens by the microalgae *Chlamydomonas mexicana*, *Scenedesmus obliquus*, *Selenastrum capricornutum*, and *Desmodesmus subspicatus*, respectively [16,35,56]. The removal and biodegradation of E2 was significantly easier than that of DES in the mixture treatments and was also higher than the value of E2 in single treatments. The difference between E2 and DES removal by microalgae may have been due to the different half-life between the natural steroidal estrogen and synthetic non-steroidal estrogen [57]. Furthermore, the presence of DES enhanced the removal of E2, which may be caused by DES stimulating the enzymes related to the degradation and transformation such as glutathione S-transferase (GST), cytochrome P450, peroxidase, etc. into research on the biodegradation of organic contaminants [22,48,49]. A similar stimulated condition was reported where the presence of E2 enhanced the removal of EE2 [17].

Hydroxylation, glycosylation, and methylation are the most generally accepted transformation processes of organic contaminant degradation by microalgae [55]. Hydroxylation, the most widespread reaction, was catalyzed by the cytochrome P450 monooxygenase (Cyt P450) for detoxification in microalgae, which could increase the polarity and hydrophilicity of the xenobiotics [58]. The products of hydroxylation might be conjugated with glucose or glutathione, which could further increase the solubility of the xenobiotics. Furthermore, the methylation products would further decrease bioavailability and may contribute to the high-efficiency elimination of contaminants [47,59]. These transformation processes were indeed efficient for the degradation of triclosan by *Desmodesmus* sp., *Chlorella pyrenoidosa*, and *Scenedesmus obliquus* [60]. E2 could be oxidized to estrone (E1), and then hydroxylated to estriol (E3) by the microalgae *Scenedesmus dimorphus* [19]. Moreover, the microalgae generated biogenic manganese oxides (BioMnOx) may promote BPA oxidation and enhance the accumulation of substrates for glycosylation [61]. Nevertheless, these known and unknown processes suggest the diverse abilities of microalgae in transforming estrogens. The pathways of degradation and transformation by microalgae will be further investigated in the future. The interactions between estrogen compounds and the differences in the removal, absorption, and transformation in mixed estrogens will also be further investigated.

Previous studies have all shown that estrogens might subsequently cause potential risks to organisms at higher trophic levels with biomagnification along food chains in aquatic ecosystems [62]. The present study showed that the microalgae *R. subcapitata* demonstrated a high capability for the removal of the E2 and DES at mg·L^−1^ levels, indicating future prospects for application in the treatment of wastewater and possibility of reducing the risk. Furthermore, microalgae could increase the degradation of contaminants in photodegradation [63] and also associate with the bacterial community to degrade the complicated contaminants [24]. It was indicated that microalgae could be associated with other techniques in increasing the removal efficiency and supplement the insufficiencies of a single technique mode in the degradation of contaminants.

## 5. Conclusions

The investigation of *Raphidocelis subcapitata* in this study showed that it could efficiently remove E2 and DES. The removal capacity of E2 was more efficient than that of DES by *R. subcapitata*. The presence of DES could accelerate the removal of E2 from the mixture treatments in equal concentrations. The removal mechanisms included initial rapid adsorption and absorption, followed by bioaccumulation and biodegradation. In addition, the removal was mainly attributed to the biodegradation or biotransformation process by the microalgae cells rather than simple sorption and accumulation in the cells. The microalgae *R. subcapitata* demonstrated a high capability for the removal of the E2 and DES at mg·L^−1^ levels, indicating future prospects its application in the treatment of wastewater. Furthermore, the interactions between estrogen compounds and the mechanisms in the removal, bioadsorption, and transformation in single and mixed estrogens should be further investigated.

## Figures and Tables

**Table 1:** The toxicity of estrogens to *R. subcapitata* (96 h growth inhibition).

Estrogens	EC_50_	95% Confidence
E2	>3.2 mg·L^−1^	-
DES	1.011 mg·L^−1^	0.79–1.22 mg·L^−1^

**Table 2:** Residual, intracellular, and extracellular E2 and DES contents under single treatments.

Treatments (mg/L)	Times (h)	Amount of Estrogens					
		Residual (μg·L^−1^)		Extracellular (10^−8^ µg·cell^−1^)		Intracellular (10^−8^ µg·cell^−1^)	
		E2	DES	E2	DES	E2	DES
0.1	24	29.2 ± 0.7 *	41.2 ± 1.1	0.31 ± 0.08	0.45 ± 0.15	0.21 ± 0.05	0.23 ± 0.08
	48	24.3 ± 1.4 *	34.3 ± 1.8	0.25 ± 0.02 *	0.85 ± 0.05	0.20 ± 0.08 *	0.43 ± 0.05
	96	17.6 ± 1.7	9.9 ± 0.8 *	0.31 ± 0.05	0.24 ± 0.05	ND *	0.15 ± 0.07
0.5	24	213.1 ± 5.5 *	268.7 ± 23.1	0.98 ± 0.24 *	6.0 ± 0.25	1.45 ± 0.33 *	2.12 ± 0.42
	48	121.7 ± 11.5 *	158.7 ± 13.3	0.88 ± 0.1 *	2.11 ± 0.67	2.26 ± 0.12 *	4.34 ± 0.15
	96	95.8 ± 10.6 *	129.5 ± 11.4	0.45 ± 0.11 *	3.33 ± 0.59	1.6 ± 0.21 *	4.10 ± 0.15
1.5	24	750.2 ± 18.1 *	861.9 ± 10.6	14.8 ± 6.6 *	18.1 ± 3.9	8.41 ± 2.1 *	14.2 ± 5.51
	48	501.5 ± 11.9 *	778.1 ± 15.5	7.89 ± 0.93 *	14.1 ± 0.75	6.32 ± 0.24 *	18.2 ± 0.51
	96	377.5 ± 16.7 *	680.3 ± 14.2	1.3 ± 0.37 *	26.52 ± 1.25	2.8 ± 0.25 *	24.2 ± 2.83

Mean and standard deviation of three replicates are shown. Asterisk (*) indicates value significantly different between the amount of E2 and DES in the same treatment (concentration and time) (*p* < 0.05).

**Table 3:** Residual, intracellular, and extracellular E2 and DES contents under mixed treatments.

Treatments (mg/L)	Times (h)	Amount of Estrogens					
		Residual (μg·L^−1^)		Extracellular (10^−8^ µg·cell^−1^)		Intracellular (10^−8^ µg·cell^−1^)	
		E2	DES	E2	DES	E2	DES
0.1	24	48.4 ± 1.7	46.7 ± 3.3	0.28 ± 0.05 *	0.48 ± 0.09	0.22 ± 0.09	0.30 ± 0.04
	48	38.2 ± 2.1	36.7 ± 2.2	0.28 ± 0.02 *	0.91 ± 0.03	0.22 ± 0.05 *	0.55 ± 0.07
	96	11.2 ± 1.3	12.6 ± 0.7	0.28 ± 0.03 *	0.31 ± 0.08	ND *	0.25 ± 0.05
0.5	24	237.8 ± 6.4 *	255.9 ± 18.5	1.08 ± 0.15 *	5.3 ± 0.71	1.61 ± 0.37	1.81 ± 0.15
	48	148.2 ± 21.3 *	182.5 ± 11.3	1.04 ± 0.2 *	3.33 ± 0.15	1.83 ± 0.37 *	5.11 ± 0.41
	96	83.7 ± 6.5 *	137.1 ± 15.5	0.21 ± 0.08 *	5.65 ± 0.88	1.5 ± 0.09 *	6.1 ± 0.29
1.5	24	711.5 ± 15.4 *	931.7 ± 13.3	3.33 ± 8.1 *	11.5 ± 2.1	6.51 ± 1.5 *	18.7 ± 3.8
	48	477.2 ± 20.5 *	822.3 ± 19.3	6.54 ± 0.66 *	15.2 ± 0.33	3.83 ± 0.85 *	11.1 ± 0.35
	96	233.9 ± 25.2 *	724.5 ± 13.3	2.5 ± 0.57 *	28.54 ± 2.55	3.8 ± 0.13 *	21.7 ± 3.65

Mean and standard deviation of three replicates are shown. Asterisk (*) indicates value significantly different between the amount of E2 and DES in the same treatment (concentration and time) (*p* < 0.05).
